# Supplementary material for: Gastro-Protective Effect of Fermented Soybean (Glycine max (L.) Merr.) in a Rat Model of Ethanol/HCl-Induced Gastric Injury
Source: Nutrients. 2022 May 16;14(10):2079. doi: 10.3390/nu14102079 (PMC9147855; doi:10.3390/nu14102079)
Supplement: Supplementary file 1 [file nutrients-14-02079-s001.zip › nutrients-1691236-supplementary.pdf]

Supplementary Materials

Gastro-Protective Effect of Fermented Soybean (*Glycine max* (L.) Merr.) in a Rat Model of Ethanol/HCl-Induced Gastric Injury

Minhee Lee[1], Dakyung Kim [1,] Hyunji Kim [2], Sukyung Jo [3], Ok-Kyung Kim[4,*] and Jeongmin Lee [1,5,*]

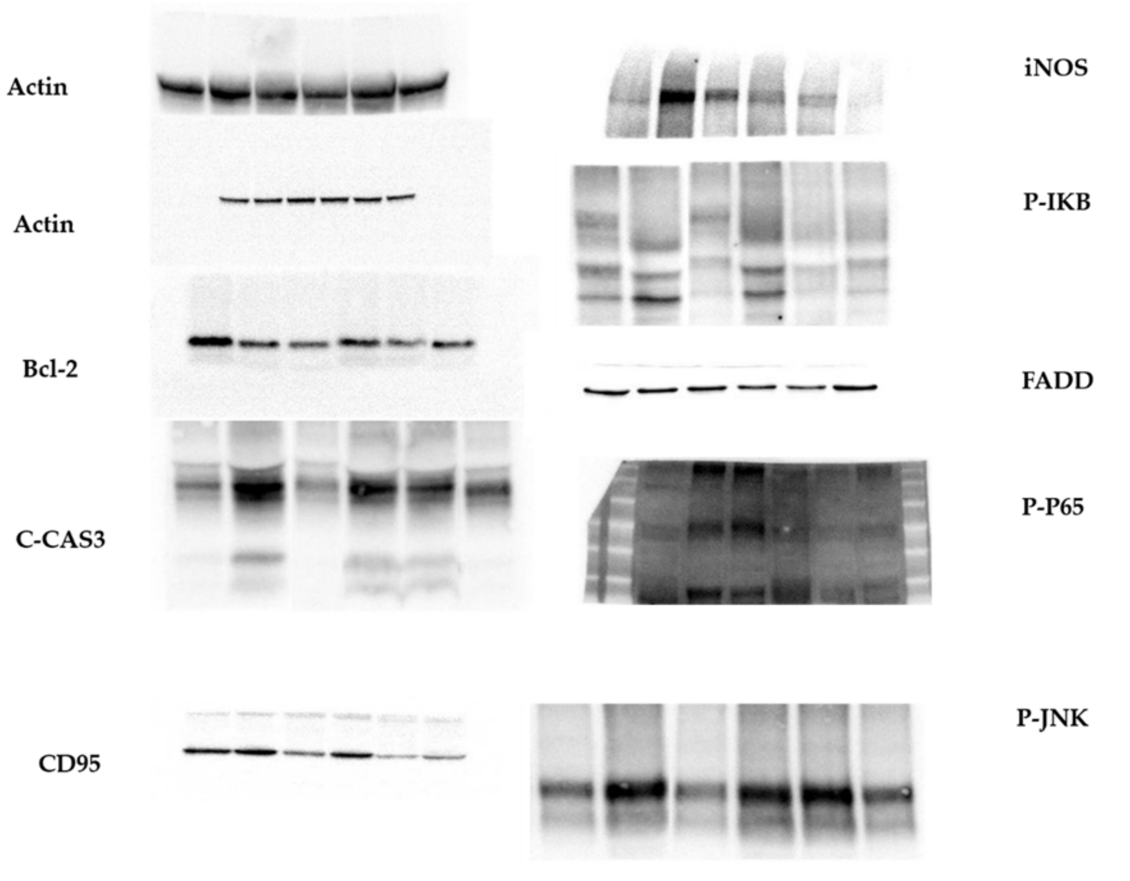

Figure S1. Expanded, uncropped western blot panels from Figure 4-5.
